# Incidence, Risk Factors, and Comorbidities of Vocal Cord Paralysis After Surgical Closure of a Patent Ductus Arteriosus: A Meta-analysis

**DOI:** 10.1007/s00246-018-1967-8

**Published:** 2018-08-24

**Authors:** Brandon Michael Henry, Wan Chin Hsieh, Beatrice Sanna, Jens Vikse, Dominik Taterra, Krzysztof A. Tomaszewski

**Affiliations:** 1 Division of Cardiology, Cincinnati Children’s Hospital Medical Center, Cincinnati, OH USA; 2 International Evidence-Based Anatomy Working Group, 12 Kopernika St 31–034 Kraków, Poland; 3 Department of Anatomy, Jagiellonian University Medical College, Kraków, Poland; 4 First Faculty of Medicine, Charles University, Prague, Czech Republic; 5 Department of Surgery, Stavanger University Hospital, Stavanger, Norway

**Keywords:** Patent ductus arteriosus, Recurrent laryngeal nerve injury, Vocal cord paralysis, Meta-analysis

## Abstract

Surgical closure of a patent ductus arteriosus (PDA) is considered standard treatment for symptomatic neonates refractory to medical therapy. Sometimes, iatrogenic injury to the left recurrent laryngeal nerve during the procedure can result in vocal cord paralysis (VCP). This study aimed to estimate the incidence of VCP in patients after surgical PDA closure and to identify any associated risk factors and morbidities associated with VCP in the preterm infant population. A thorough search of the major electronic databases was conducted to identify studies eligible for inclusion into this meta-analysis. Studies reporting data on the incidence of VCP (primary outcomes) or risk factors and morbidities associated with VCP in premature infant population (secondary outcomes) were included. A total of 33 studies (*n* = 4887 patients) were included into the analysis. Overall pooled incidence estimate of VCP was 7.9% (95%CI 5.3–10.9). The incidence of VCP after PDA closure was significantly much higher in premature infants (11.2% [95%CI 7.0–16.3]) than in non-premature patients (3.0% [95%CI 1.5–4.9]). The data showed that VCP was most common after surgical ligation and in studies conducting universal laryngoscopy scoping. The risk factors for postoperative VCP in preterm infants included birth weight and gestational age. In addition, VCP was significantly associated with the occurrence of bronchopulmonary dysplasia, gastrostomy tube insertion, and increased duration of mechanical ventilation. Vocal cord paralysis remains a frequent complication of surgical closure of a PDA, especially in premature neonates, and is associated with significant post-procedural complications.

**Electronic supplementary material:**

The online version of this article (10.1007/s00246-018-1967-8) contains supplementary material, which is available to authorized users.

## Introduction

The normal ductus arteriosus arises from the left pulmonary artery and connects to the transition area between the aortic arch and descending aorta just distal to the origin of the left subclavian artery. Physiologically, ductus arteriosus closes shortly after birth to facilitate proper breathing in the infant. Rarely it narrows and occludes prematurely during fetal life. It is estimated that 8 out of 1000 preterm infants will develop a patent ductus arteriosus (PDA), a condition in which the ductus arteriosus fails to close postnatally [1].

The presence of PDA is reported to contribute to the development of feeding intolerance, necrotising enterocolitis, intracranial hemorrhage, decreased glomerular filtration rate, and bronchopulmonary dysplasia (BPD) in preterm infants [2]. Initial treatment of PDA is usually pharmacological and includes indomethacin. Surgical closure is considered a standard treatment for symptomatic neonates refractory to medical therapy [3, 4]. During the surgical procedure, the perineurium of the left recurrent laryngeal nerve may be disrupted or the nerve may be contused or injured by the clip or ligature [5]. A possible adverse event of such an injury is vocal cord paralysis (VCP). Duration of vocal cord dysfunction is variable and may be transient (< 1 year) or persistent (> 1 year) [6].

Though VCP following PDA surgical closure is well reported in the literature, the recorded incidence of this postoperative complication varies widely, ranging from > 1 to 64% [7, 8]. Previous research provided inconclusive data on its incidence due to different methodology of assessment of VCP and variable laryngoscopy scoping. As such, there was a need for a study that would unify the existing data on the incidence of VCP based on the method used to close PDA, method to assess VCP, and evaluate the influence of age and ethnic origin on patient outcome. Therefore, the objective of this meta-analysis was to estimate the incidence of VCP after surgical PDA closure in different age populations (primary outcomes), as well as identify associated risk factors and morbidities for preterm infants (secondary outcomes).

## Materials and Methods

### Search Strategy

A search of the major electronic databases, including PubMed, ScienceDirect, EMBASE, BIOSIS, SCIELO, CNKI, and Web of Science, was performed in order to identify potential studies for inclusion in the meta-analysis. No date limits or language restrictions were applied. Search terms included patent ductus arteriosus and a selection of one of the following: recurrent laryngeal nerve or vocal cord paralysis or vocal cord paresis or vocal fold dysfunction. The references of the included studies were also searched in order to identify additional articles. The authors strictly adhered to the Preferred Reporting Items for Systematic Reviews and Meta-Analyses (PRISMA) guidelines (see Supplementary material 1).

### Study Selection Criteria

Studies were included into the meta-analysis if (1) reported clear, extractable data on the incidence of the VCP after PDA closure, (2) the study clearly defined VCP as unilateral vocal cord immobility, and (3) used clip or ligation technique for PDA closure. Meta-analysis of risk factors in preterm infants additionally included the following criteria (1) study population included preterm infants that underwent surgery for PDA, (2) outcomes reported included incidence of VCP risk factors and associated comorbidities. The following exclusion criteria were employed: (1) case studies, case reports, conference abstracts, and letters to the editor, (2) studies reporting incomplete data. Two independent reviewers assessed the eligibility of the articles for the inclusion into the meta-analysis. Any discrepancies among reviewers were resolved by a consensus among the entire review team. In case of studies published in languages other than English, medical professionals fluent in English and the original language of the article translated the texts.

### Data Extraction and Quality Assessment

The following data were extracted from the eligible studies: year, country, type of study and technique of PDA closure (suture ligature or clipping), sample size (number of patients), incidence of VCP, length of injury—transient or permanent (transient VCP was defined as lasting less than a year, while persistent as more than a year), patients’ characteristics (age, geographical origin). Patients were divided into two groups—premature newborns/low birth weight newborns and non-premature patients (any patients that did not fit into first group). Data from any study comparing different cohorts of patients (i.e., ligation vs. clip, premature vs. non-premature patients) were extracted and treated as two separate studies for the purpose of analysis. Whenever possible, outcomes comparing VCP and non-VCP groups in premature/low birth weight patients were also extracted. These included the following: birth weight, weight at the time of surgery, age (days) at the time of surgery, gestational age, incidence of bronchopulmonary dysplasia, total days of mechanical ventilation, and incidence of gastrostomy tube insertion. Critical Appraisal of the Health Research Literature tool was used to assess the quality of studies reporting the prevalence or incidence of a health problem [9]. Study quality was rated on a scale from 1 (very poor) to 8 (high). In case of any discrepancies in the data of included studies, the review team attempted to contact the authors of the original study for clarification.

### Statistical Analysis

Pooled incidence estimates were calculated using random effects model with MetaXL version 5.3 by EpiGear International Pty Ltd (Wilston, QLD, Australia). Subgroup analysis based on type of PDA closure and geographical distribution of the studies was performed to probe the source of heterogeneity. Confidence intervals were used to compare the incidence rates, with any overlap between two groups indicating a lack of statistical significance.

Continuous outcomes in risk factors analysis were analyzed by calculating weighted mean difference (WMD) with the 95% confidence intervals (95%CI). Dichotomous outcomes were analyzed by calculating relative risk (RR) with the 95%CI. Reported values were two-tailed, and hypothesis-testing results were considered statistically significant at *p* < 0.05.

Heterogeneity among included studies was assessed with the *χ*^2^ test and the *I*^2^ statistic. A Cochran’s Q *p* value of < 0.10 indicated significant heterogeneity [10]. The following intervals were used to analyze heterogeneity in *I*^2^ statistic: 0–40%—might not be important; 30–60%—might indicate moderate heterogeneity; 50–90%—may indicate substantial heterogeneity; and 75–100% may represent considerable heterogeneity [10].

## Results

### Literature Search

**Fig. 1:**
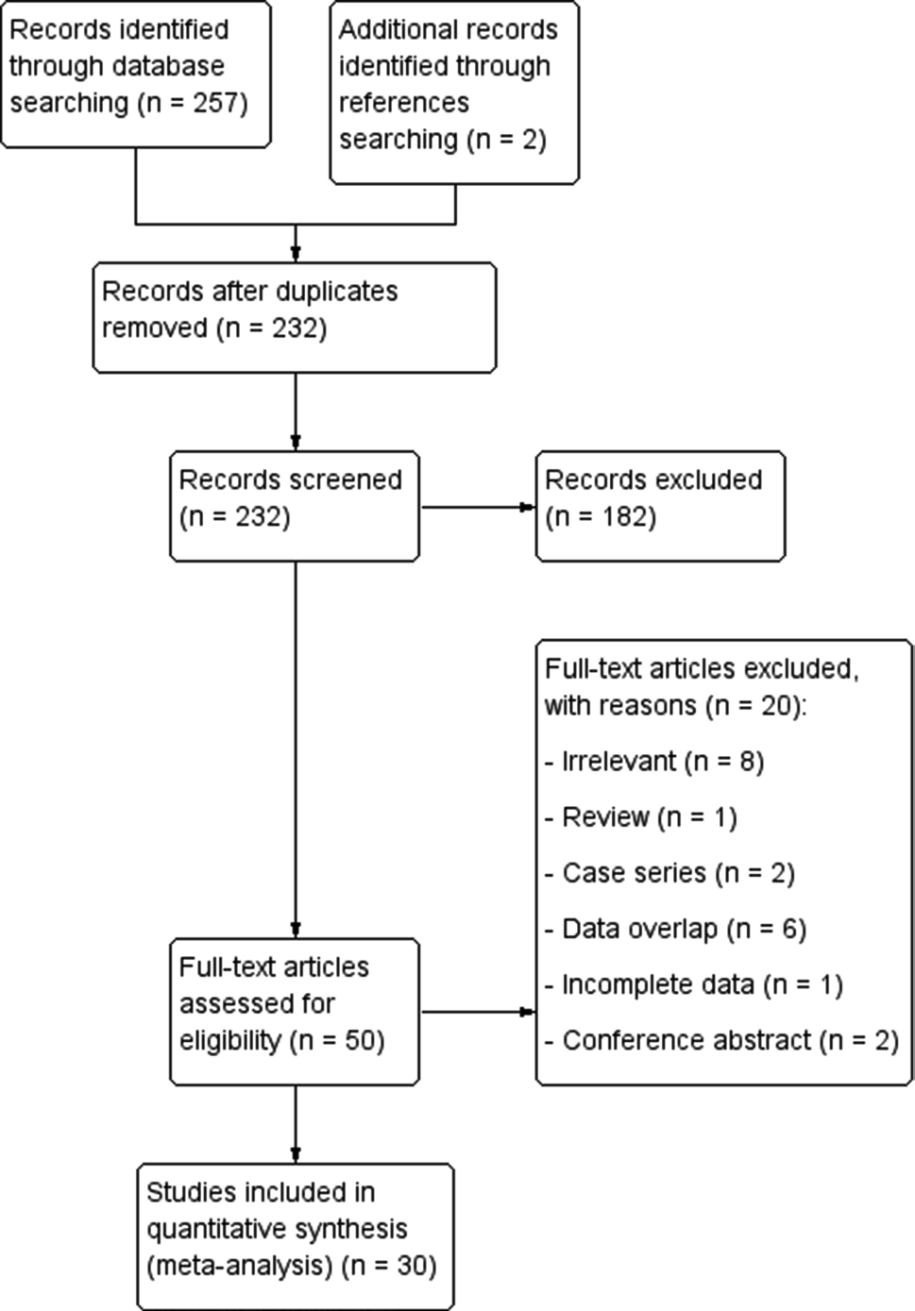
Study identification flowchart

The study identification flow chart is presented in Fig. 1. The initial electronic search yielded 257 potential articles. Additional two articles were identified through reference search. After eligibility analysis, 30 articles were included in this meta-analysis.

### Characteristics of the Eligible Studies

The characteristics of included studies with quality assessment are presented in Table 1. Due to the fact that three articles [11–13] compared two different cohorts of patients, each cohort was treated as a separate study, therefore 33 studies (*n* = 4887 subjects) were included into this meta-analysis. Specifically, the study by Davis et al. [11] contained two groups of patients, one treated by ligation and one by clip method, and thus for the purposes of analysis was considered as two separate studies. The study by Spanos et al. [12] was treated as two separate studies for the same reason. The study by Villa et al. [13] analyzed two groups of patients (low birth weight and non-premature patients), thus it was also treated as two separate studies during analysis.

**Table 1:** Characteristics of included studies

Study ID	Country	Study design	Type of PDA closure	Method to assess VCP	Premature	Number of patients	Incidence of VCP (%)	Study quality
Benjamin et al. [5]	USA	Prospective	Ligation	Selective scoping^b^	Yes	55	40.0	7
Bensky et al. [14]	USA	Prospective	Clip	Selective scoping	Yes/No	118	2.5	6
Burke et al. [15]	USA	Prospective	Ligation	Symptoms only^a^	Yes	34	2.9	6
Carpes et al. [16]	Canada	Prospective	Ligation	Pre-op and post-op universal scoping	No	42	16.7	6
Clement et al. [17]	Canada	Retrospective	Ligation	Universal scoping^c^	Yes	23	52.2	6
Davis et al. [11]	USA	Retrospective	Ligation	Selective scoping	Yes	68	4.4	5
Davis et al. [11]	USA	Retrospective	Clip	Selective scoping	Yes	38	0.0	5
Ekici et al. [18]	Turkey	Retrospective	Ligation	Symptoms only	Yes	12	8.3	6
Hawkins et al. [36]	USA	Retrospective	Ligation	Symptoms only	No	20	5.0	5
Heuchan et al. [19]	England	Retrospective	Ligation	Symptoms only	Yes	125	4.8	6
Hines et al. [20]	USA	Retrospective	Clip	Selective scoping	Yes	100	5.0	6
Ibrahim et al. [21]	Egypt	Retrospective	Ligation	Symptoms only	Yes	120	0.8	5
Kang et al. [22]	England	Prospective	Ligation	Symptoms only	Yes	102	2.0	6
Laborde et al. [23]	France	Retrospective	Clip	Symptoms only	No	332	1.8	6
Liem et al. [24]	Vietnam	Prospective	Clip	Symptoms only	No	58	0.0	5
Mandhan et al. [25]	New Zealand	Retrospective	Ligation and clip	Symptoms only	Yes	145	0.7	6
Nezafati et al. [26]	Iran	Retrospective	Clip	Symptoms only	No	1300	1.0	6
Nichols et al. [27]	USA	Retrospective	Ligation	Selective scoping	Yes	532	12.4	5
Niinikoski et al. [3]	Finland	Retrospective	Surgical	Symptoms only	Yes	101	1.0	5
Odegard et al. [28]	USA	Prospective	Clip	Symptoms only	No	60	1.7	5
Pereira et al. [29]	USA	Prospective	Ligation	Universal scoping	Yes	61	11.5	5
Pharande et al. [30]	Australia	Retrospective	Ligation	Selective scoping	Yes	35	31.4	6
Roksund et al. [8]	Norway	Prospective	Surgical	Universal scoping	Yes	11	63.6	7
Rukholm et al. [31]	Canada	Retrospective	Ligation	Only 31/111 patients had LAR post-op	Yes	111	17.1	7
Smith et al. [32]	USA	Prospective	Ligation	Universal scoping	Yes	86	16.3	6
Sørensen et al. [33]	Denmark	Retrospective	Ligation	Symptoms only	Yes	46	6.5	5
Spanos et al. [13]	USA	Prospective	Ligation	Universal scoping	Yes	41	19.5	6
Spanos et al. [13]	USA	Prospective	Clip	Universal scoping	Yes	27	18.5	6
Vanamo et al. [34]	Finland	Prospective	Ligation	Symptoms only	No	110	6.4	5
Vida et al. [35]	Italy	Prospective	Clip	Symptoms only	No	150	0.7	6
Villa et al. [13]	France	Retrospective	Clip	Selective scoping	No	721	2.5	5
Villa et al. [13]	France	Retrospective	Clip	Selective scoping	Yes	22	13.6	5
Zbar et al. [4]	USA	Retrospective	Ligation	Selective scoping	No	81	7.4	6

^a^Vocal cord paralysis was diagnosed based on symptoms only

^b^Only symptomatic patients had laryngoscopy postoperatively to confirm vocal cord paralysis

^c^All patients were scoped with laryngoscopy postoperatively

In total, fourteen studies were prospective and the other nineteen were retrospective. Ligation was the most frequently used surgical closure approach. Only one study analyzed all patients with laryngoscopy preoperatively. A total of seven studies scoped all patients postoperatively with laryngoscopy to assess for VCP. All of those studies used a surgical approach and 6/7 analyzed premature patients. Ten studies utilized selective scoping with laryngoscopy of patients that showed symptoms suggestive of VCP. Sixteen studies identified patients with VCP based only on symptoms or method was not mentioned.

### Overall Incidence of VCP

**Fig. 2:**
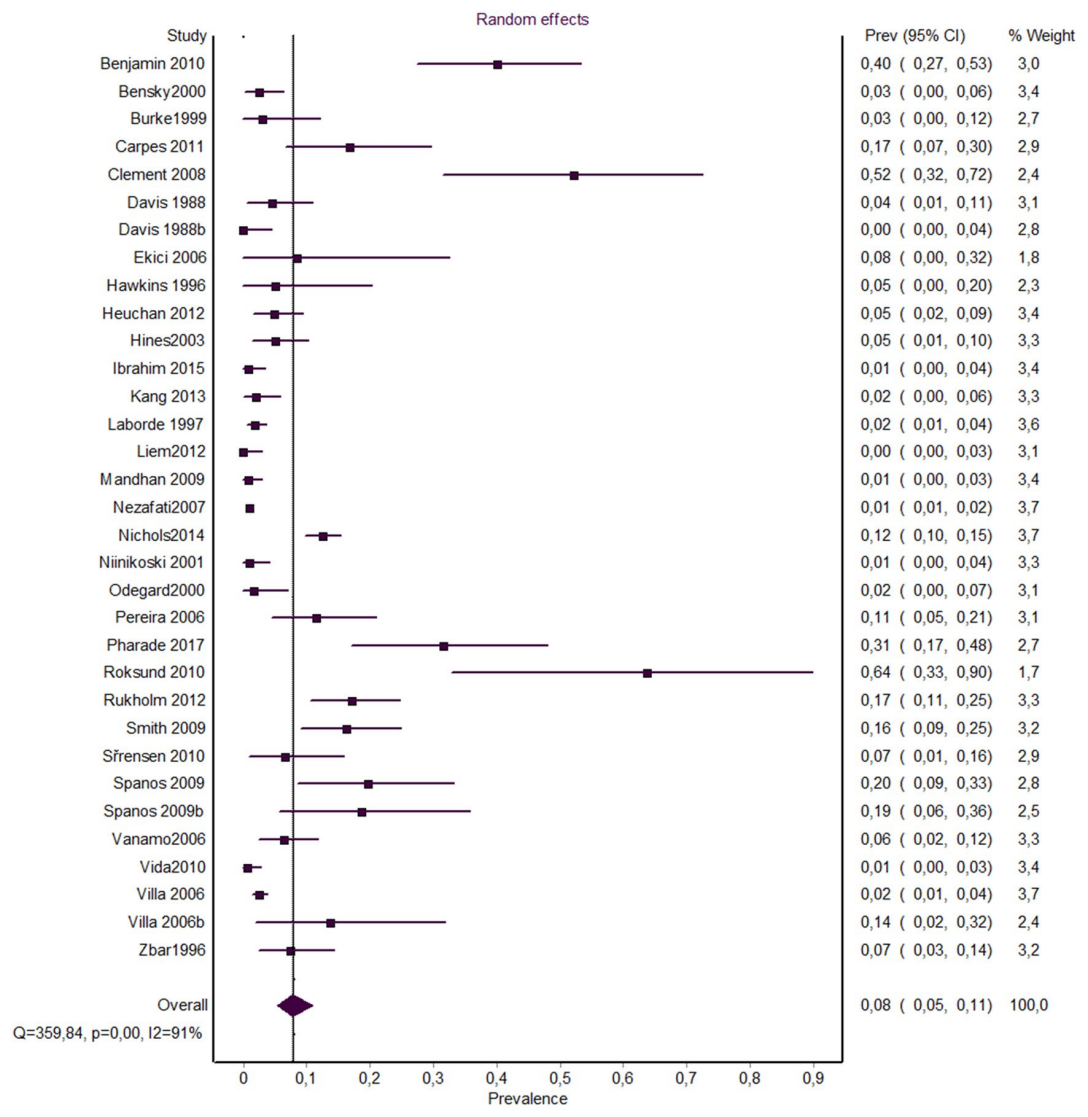
Forest plot for overall pooled incidence rate of vocal cord paralysis

A total of 33 studies (*n* = 4887 subjects) reported extractable data on the incidence of VCP in all age populations after PDA closure (Table 2). Overall pooled incidence estimate of VCP was 7.9% (95%CI 5.3–10.9) (Fig. 2).

**Table 2:** Overall incidence of vocal cord paralysis after patent ductus arteriosus closure

Subgroup	Number of studies (number of subjects)	Pooled incidence of VCP after PDA closure: % (95% CI)	*I*^2^ (%)	Cochran’s Q, *p* value
Overall	33 (4887)	7.9 (5.3–10.9)	91.1	< 0.001
Method of PDA closure				
Surgical ligation	20 (1805)	11.1 (7.2–15.8)	86.5	< 0.001
Surgical clipping	11 (2926)	2.4 (1.3–3.9)	69.0	0.001
Method to assess for VCP				
Universal laryngoscopy scoping^a^	7 (291)	24.2 (14.5–35.3)	75.0	0.001
Selective laryngoscopy scoping^b^	10 (1770)	9.1 (4.2–15.6)	92.4	< 0.001
Symptoms scoping^c^	15 (2715)	2.2 (1.3–3.3)	50.3	0.013
Geographical origin				
Asia	2 (1358)	1.0 (0.5–1.6)	0.00	0.582
Europe	11 (1732)	4.5 (2.3–7.3)	77.7	< 0.001
North America	17 (1497)	11.5 (7.4–16.5)	84.5	< 0.001

*PDA* patent ductus arteriosus, *VCP* vocal cord paralysis

^a^All patients were scoped with laryngoscopy postoperatively to assess for VCP

^b^Only symptomatic patients had laryngoscopy to assess for VCP

^c^VCP was diagnosed only based on symptoms

VCP occurred most often in patients treated with surgical ligation (11.1%; [95%CI 7.2–15.8]). Surgical clipping caused significantly less VCP than surgical ligation, with pooled VCP incidence rate of 2.4% (95%CI 1.3–3.9). Analysis of studies assessing all patients postoperatively with laryngoscopy (24.2% [95%CI 14.5–35.3]) showed significantly higher pooled incidence of VCP than studies that diagnosed VCP based only on symptoms (2.2% [95%CI 1.3–3.3], Table 2) or studies assessing only symptomatic patients (9.1% [95%CI 4.2–15.6]).

Geographical analysis revealed that VCP after PDA closure occurred more often in North America (11.5% [95%CI 7.4–16.5]) than in Europe (4.5% [95%CI 2.3–7.3]) or Asia (1.0% [95%CI 0.5–1.6]).

### Incidence of VCP in Premature/Low Birth Weight Patients

Twenty-two studies (*n* = 1895 patients) were included in the analysis on the incidence of VCP after PDA closure in premature newborns (Table 3). The overall pooled incidence of VCP in this population was 11.2% (95%CI 7.0–16.3). Premature infants were more likely to develop VCP after surgical ligation (11.7%; [95%CI 6.9–17.5]) than surgical clipping (6.8%; [95%CI 0.7–16.9]). Subgroup analysis on geographical origin showed that VCP in premature patients seems to occur more frequently in North America (14.0%; [95%CI 8.6–20.5]) than Europe (8.2%; [95%CI 2.5–16.4]).

**Table 3:** Incidence of vocal cord paralysis after patent ductus arteriosus closure in premature /low birth weight patients

Subgroup	Number of studies (number of subjects)	Pooled incidence of VCP after PDA closure: % (95% CI)	*I*^2^ (%)	Cochran’s Q, *p* value
Overall	22 (1895)	11.2 (7.0–16.3)	89.2	< 0.001
Method of PDA closure				
Surgical ligation	16 (1552)	11.7 (6.9–17.5)	89.0	< 0.001
Surgical clipping	4 (187)	6.8 (0.7–16.9)	73.5	0.010
Geographical origin				
Europe	7 (419)	8.2 (2.5–16.4)	81.3	< 0.001
North America	12 (1176)	14.0 (8.6–20.5)	85.3	< 0.001

*PDA* patent ductus arteriosus, *VCP* vocal cord paralysis

### Incidence of VCP in Non-premature Patients

Ten studies (*n* = 2874 patients) reported extractable data on the incidence of VCP after PDA closure in non-premature patients (Table 4). The overall pooled incidence estimate in this population was 3.0% (95%CI 1.5–4.9). Again, surgical ligation resulted in significantly higher incidence of VCP (8.6%; [95%CI 5.0–13.0]) than surgical clipping (1.5%; [95%CI 0.9–2.3]). Subgroup analysis on geographical origin showed that VCP in non-premature patients seems to occur more frequently in North America (7.1% [95%CI 2.2–14.4]) than other regions.

**Table 4:** Vocal cord paralysis after patent ductus arteriosus closure in non-premature patients

Subgroup	Number of studies (number of subjects)	Pooled incidence of VCP after PDA closure: % (95% CI)	*I*^2^ (%)	Cochran’s Q, *p* value
Overall	10 (2874)	3.0 (1.5–4.9)	77.7	< 0.001
Method of PDA closure				
Surgical clipping	6 (2621)	1.5 (0.9–2.3)	59.4	0.043
Surgical ligation	4 (253)	8.6 (5.0–13.0)	18.3	0.299
Geographical origin				
Asia	2 (1358)	1.0 (0.5–1.6)	0.00	0.582
Europe	4 (1313)	2.4 (1.1–4.1)	60.0	0.059
North America	4 (203)	7.1 (2.2–14.4)	60.7	0.054

*PDA* patent ductus arteriosus, *VCP* vocal cord paralysis

### Duration of Vocal Cord Paralysis

Overall, 10 studies (*n* = 121 patients with VCP) analyzed the duration of VCP after PDA closure. A total of 72.3% (95%CI 30.8–100.0; *I*^2^ = 92.0; *p* < 0.001) of patients developed transient VCP. In premature newborns (five studies, 82 patients with VCP), the majority (64.9%; 95%CI 20.2–100.0%; *I*^2^ = 84.5; *p* < 0.001) suffered from persistent VCP. Lastly, an analysis of six studies (*n* = 46 patients with VCP) on non-premature patients showed that these patients more often had transient VCP (83.4%; 95%CI 71.5–92.8%; *I*^2^ = 0.0; *p* = 0.631).

### Risk Factors of VCP

#### Birth Weight

Six studies (*n* = 321 subjects) compared the birth weight between preterm infants with VCP and those without VCP (Table 5). It should be noted that birth weight in preterm infants with VCP was significantly lower than those without VCP (WMD = − 149.03 g; 95%CI − 269.02 to − 29.05 g; *p* = 0.02).

**Table 5:** Risk factors and comorbidities in premature infants/low birth weight infants for vocal cord paralysis after patent ductus arteriosus closure

Risk factor/comorbidity	Number of studies included (number of patients)	Type of outcome calculation	Value of outcome analysis (95% CI)	*p* value	*I*^2^ (%)	Cochran’s Q, *p* value
Birth weight	6 (321)	WMD	− 149.03 g (− 269.02 to − 29.05)	0.02	90.3	< 0.001
Gestational age	6 (321)	WMD	− 1.21 week (− 1.79 to − 0.63)	< 0.01	64.1	0.016
Weight at ligation	4 (287)	WMD	− 258.00 g (− 548.15 to 32.15)	0.08	96.8	< 0.001
Days of life at ligation	4 (224)	WMD	− 8.96 days (− 26.17 to 8.25)	0.31	98.0	< 0.001
Bronchopulmonary dysplasia	4 (200)	RR	1.23 (1.00− 1.51)	0.049	0.0	0.974
Duration of mechanical ventilation	3 (89)	WMD	16.16 days (4.24 to 28.08)	< 0.01	58.6	0.089
Gastrostomy tube insertion	3 (224)	RR	1.22 (1.00 to 1.49)	0.03	57.8	0.069

*CI* confidence interval, *RR* relative risk, *WMD* weighted mean difference

#### Gestational Age

Six studies (*n* = 321 subjects) reported the gestational age of preterm infants with and without VCP. As shown in Table 5, gestational age in preterm infants with VCP was significantly lower than those without VCP (WMD = − 1.21 weeks; 95%CI − 1.79 to − 0.63 weeks; *P* < 0.01).

#### Weight at Ligation

Four studies (*n* = 287 subjects) compared the weight at ligation between preterm infants with and without VCP. Pooled results in Table 5 indicate that weight at ligation in preterm infants with VCP was lower than those without VCP (WMD = − 258.00 g; 95%CI − 548.15 to − 32.15 g; *p* = 0.08); however, the difference was not significant.

#### Days of Life at Ligation

Four studies (*n* = 224 subjects) compared the days of life at ligation between preterm infants with and without VCP. Table 5 shows that the total days of life at ligation in preterm infants with VCP was shorter than those without VCP (WMD = − 8.96 days; 95%CI − 26.17 to 8.25 days; *p* = 0.31); however, the difference was not significant.

### Comorbidities of VCP

#### Bronchopulmonary Dysplasia

There was slight significant difference in the incidence of BPD in preterm infants with and without VCP, indicating that preterm infants with VCP were mildly more likely to suffer from BPD (four studies, *n* = 200 subjects; RR = 1.23; 95%CI 1.00–1.51; *p* = 0.049; I^2^ = 0.0, Table 5).

#### Duration of Mechanical Ventilation

The effect estimate revealed a significant difference in total days of mechanical ventilation between preterm infants with and without VCP (Table 5), showing that preterm infants with VCP needed prolonged mechanical ventilation (three studies, *n* = 89 patients; WMD = 16.16 days; 95%CI 4.24–28.08 days; *p* < 0.01).

#### Gastrostomy Tube Insertion

Meta-analysis of three studies (*n* = 224 subjects) showed that there was slight significant difference in the incidence of gastrostomy tube insertion between preterm infants with and without VCP, indicating that preterm infants with VCP were more likely to require gastrostomy tube insertion (RR = 1.22; 95%CI 1.00–1.49; *p* = 0.03; Table 5).

## Discussion

The ductus arteriosus is indispensable in the maintenance of fetal circulation. Failure to close in the first few days after birth may result in high morbidity and mortality if not treated correctly. Surgery is the standard method of closure in approximately a quarter of extremely low birth weight infants, as reported by the National Institute of Child Health and Human Development Neonatal Research Network [37]. Though the advantages of surgical closure of PDA and its short- and long-term efficacy and safety have been highlighted, multiple studies have demonstrated that VCP remains a common complication of these procedures. This meta-analysis assessed the incidence of VCP in various age populations and evaluated its risk factors and comorbidities in preterm infants following interventional closure of PDA.

To date, the true incidence of VCP after interventional PDA closure has been unclear. Our meta-analysis involving 4887 patients from 33 studies showed that the total incidence of VCP was (7.9% [95%CI 5.3–10.9]). The incidence of VCP after PDA closure was significantly higher in premature infants (11.2% [95%CI 7.0–16.3]) than in non-premature patients (3.0% [95%CI 1.5–4.9]), indicating premature infants are at a greater risk of VCP. Truong et al.’s study [6] illustrated that compared with term infants, preterm infants are less likely to recover vocal cord function. It could also be demonstrated in our study as persistent VCP occurred more frequently in premature newborns, while transient VCP was more commonly found in non-premature patients. A lower incidence of VCP after surgical clipping was found in this study, along with shorter operative time and lower risk of bleeding [25], should effectively encourage the use of this technique of surgical PDA closure in appropriately qualified patients.

The method of assessing and diagnosing VCP is of crucial importance in discerning the true incidence of this complication. The analysis of studies that scoped all patients postoperatively with laryngoscopy, regardless of the presence of laryngeal symptoms, showed significantly higher incidence of VCP (24.2%) as compared to studies that assessed only symptomatic patients (7.5%) or based diagnosis of VCP on symptoms alone (2.0%). Though the majority of universal scoping studies were done on premature patients, which had a higher incidence of VCP to begin with, these findings suggest that VCP might be highly underestimated. This is particularly evident by the low pooled incidence of VCP in studies diagnosing patients only based on symptoms. Geographical differences may be partially attributed to the lack of studies conducting universal laryngoscopy scoping in Europe and Asia, thus underestimating the incidence of VCP in those continents.

Birth weight and gestational age were significantly associated with the occurrence of VCP. Even though the difference was not significant, the weight at ligation and days of life at ligation in preterm infants with VCP were lower than those without VCP. It is not unexpected, as the smaller and weaker infants are more likely to suffer from complication PDA surgical closure.

Preterm infants with VCP were more likely to have respiratory problems. The exact causal relationship between VCP and BPD remains unclear. Chronic microaspiration due to impaired airway protection following extubation could be a mechanism causing ongoing lung injury; however, it also is possible that infants with the most-severe lung disease are most likely to undergo surgical treatment of PDA [17]. Along with the development of BPD, the functional residual capacity has been shown to be reduced, which requires glottal closure to create positive-end expiratory pressure. Extubated infants with VCP and secondary glottic incompetence may experience decreased pulmonary function, leading to prolonged mechanical support to maintain functional residual capacity. Moreover, the high risk of aspiration for infants with VCP may induce lung injury and contribute to a prolonged duration of mechanical ventilation.

Swallowing difficulties are common manifestation of vocal cord dysfunction after cardiothoracic surgery, leading to significant feeding problems [38]. Compared with infants who did not have symptoms of VCP following PDA surgery, those with VCP were more likely to have ongoing problems with feeding and growth, thereby requiring total or supplemental tube feedings at hospital discharge.

Several limitations of this meta-analysis should be considered. First, despite the high incidence of VCP in the preterm infants following PDA surgery, there remains a lack of coordinated, high-quality studies in the literature. In addition, many included studies are limited by their retrospective design. The incidence of VCP may be highly underestimated because most asymptomatic patients did not undergo laryngoscopy and a large proportion of patients were diagnosed based on symptoms alone.

## Conclusions

We report an overall VCP pooled incidence of 6.9% and high pooled incidence of VCP in premature infants (11.7%). The data showed that VCP was most common after surgical ligation and was the highest in studies conducting universal laryngoscopy scoping. The risk factors for postoperative VCP in preterm infants included birth weight and gestational age. In addition, VCP was associated with the occurrence of BPD and gastrostomy tube insertion, as well as an increased duration of mechanical ventilation.

## Electronic supplementary material

Below is the link to the electronic supplementary material.

Supplementary material 1 (DOC 63 KB)
